# Galectin-3 as a Biomarker for Stratifying Abdominal Aortic Aneurysm Size in a Taiwanese Population

**DOI:** 10.3389/fcvm.2021.663152

**Published:** 2021-05-31

**Authors:** Hsin-Ying Lu, Chun-Ming Shih, Shih-Hsien Sung, Alexander T. H. Wu, Tsai-Mu Cheng, Yen-Chung Lin, Chun-Che Shih

**Affiliations:** ^1^Division of Cardiovascular Surgery, Department of Surgery, Wan Fang Hospital, Taipei Medical University, Taipei, Taiwan; ^2^Institute of Clinical Medicine, National Yang Ming Chiao Tung University, Taipei, Taiwan; ^3^Taipei Heart Institute, Taipei Medical University, Taipei, Taiwan; ^4^Division of Cardiology, Department of Internal Medicine, Taipei Medical University Hospital, Taipei, Taiwan; ^5^Department of Internal Medicine, School of Medicine, College of Medicine, Taipei Medical University, Taipei, Taiwan; ^6^Division of Cardiology, Department of Medicine, Taipei Veterans General Hospital, Taipei, Taiwan; ^7^Department of Medicine, School of Medicine, National Yang Ming Chiao Tung University, Taipei, Taiwan; ^8^The Ph.D. Program for Translational Medicine, College of Medical Science and Technology, Taipei Medical University, Taipei, Taiwan; ^9^Division of Nephrology, Department of Internal Medicine, Taipei Medical University Hospital, Taipei, Taiwan; ^10^Department of Surgery, School of Medicine, College of Medicine, Taipei Medical University, Taipei, Taiwan

**Keywords:** galectin-3, interlukin-6, abdominal aortic aneurysm, inflammation, biomarker

## Abstract

Abdominal aortic aneurysm (AAA) ruptures are unpredictable and lethal. A biomarker predicting AAA rupture risk could help identify patients with small, screen-detected AAAs. Galectin-3 (Gal-3), a β-galactosidase–binding lectin, is involved in inflammatory processes and may be associated with AAA incidence. We investigated whether Gal-3 can be used as a biomarker of AAA size. Plasma Gal-3 protein concentrations were examined in patients with AAA (*n* = 151) and control patients (*n* = 195) using Human ProcartaPlex multiplex and simplex kits. Circulating Gal-3 levels were significantly higher in patients with AAA than in control patients. The area under the receiver operating characteristic curve for Gal-3 was 0.91. Multivariate logistic regression analysis revealed a significant association between Gal-3 level and the presence of AAA. Circulating Gal-3 levels were significantly correlated with aortic diameter in a concentration-dependent manner. In conclusion, higher plasma Gal-3 concentrations may be a useful biomarker of AAA progression.

## Introduction

Abdominal aortic aneurysm (AAA) can cause segmental expansion and rupturing of the aorta, which are common among older adults and potentially life threatening (1). Common risk factors for AAA are male sex, older age, smoking, and hypertension (2). AAAs are characterized by the destruction of elastin and collagen in the media and adventitia, loss of smooth muscle cells (SMCs), thinning of the medial wall, infiltration of lymphocytes and macrophages, and neovascularization (3). Unclear pathological mechanisms have hindered the development of effective therapeutic strategies related to AAA. At present, surgical intervention is the only treatment option. A biomarker of AAA growth and subsequent rupture risk would help in patient selection for aneurysm repair. However, no reliable markers have been identified.

AAA pathogenesis plays a major role in tissue-destructive inflammation, which involves the accumulation of inflammatory cells in the adventitia through the recruitment of circulating monocytes or the proliferation of resident macrophages (4). Inflammatory biomarkers, such as C-reactive protein, tumor necrosis factor-α, interleukin (IL)-6, IL-β, and interferon-γ, are mediators that are reportedly involved in AAA development (5, 6). A meta-analysis of case–control studies suggested that AAAs are associated with high circulating IL-6 levels (7). Furthermore, numerous members of the cysteinyl cathepsin and matrix metalloproteinase (MMP) subfamilies are potent elastases and collagenases that mediate the degradation of these extracellular matrix proteins, causing AAA expansion and rupture (8, 9). Changes in systemic cytokine levels may activate cells in the periphery and initiate complex machinery, causing the recruitment of immune cells to the aneurysmal lesion.

Galectin-3 (Gal-3), a β-galactoside–binding lectin, is characterized by a conserved sequence within the carbohydrate recognition domain and amino-terminal tandem repeats (10). It regulates chemotaxis and inflammation (11, 12). Gal-3 has been reported as a prognostic marker for cardiovascular disease as it is linked to myocardial fibrosis, tissue remodeling, and heart failure development (13, 14) as well as heart failure severity (15, 16). Plasma Gal-3 levels were reported to be markedly higher in patients with coronary artery disease (17). Fernandez-García et al. suggested that increased Gal-3 levels are associated with AAA because of the CCL5 expression caused by STAT3 activation (18).

Inflammation caused by macrophage activation is a pathological marker of AAA progression. Although Gal-3 is reportedly associated with AAA, little is known about its potential application as a biomarker for AAA. While IL-6, as well as Gal-3, is an inflammatory mediator. IL-6 has a critical role in elevating the circulating concentrations of several plasma proteins, including fibrinogen and C-reactive protein (CRP). Therefore, IL-6 has been identified as an essential pro-inflammatory cytokine in the pathogenesis of AAA (7, 19, 20). However, the prognostic value of IL-6 is controversial. The present study aimed to characterize the potential prognostic value of Gal-3. Our results indicated that Gal-3 was an independent variable for identifying subaneurysmal aortic dilatation and large aneurysms compared with IL-6.

## Materials and Methods

### Patient Recruitment

Plasma samples were collected from 151 patients diagnosed as having an AAA by computed tomography who underwent open surgical repair or endovascular aneurysm repair (EVAR) or those diagnosed as having subaneurysmal aortic dilatation at the Taipei Veterans General Hospital between 2016 and 2018. AAA and subaneurysmal aortic dilatation were diagnosed through ultrasound examination. Other inclusion criteria for patients with AAA were aortic diameter of ≥30 mm and age of 20–80 years. Patients with dissecting, infectious, traumatic, or inflammatory AAA and those with coexisting malignant tumors were excluded. The control group comprised 195 patients who regularly visited the cardiology clinic and had a normal aortic diameter (infrarenal aortic diameter of <30 mm) without a history of ischemic heart disease or renal insufficiency. The study patients were divided into three groups based on aortic diameter: control (infrarenal aortic diameter, <30 mm), large AAA (LAA, infrarenal aortic diameter, ≥55 mm), and small AAA (SAA, infrarenal aortic diameter, 30–55 mm) groups. We used G^*^power to determine the sample size with 0.99. Information on smoking status, hypertension, dyslipidemia, diabetes mellitus, chronic obstructive pulmonary disease, and use of β-blockers, angiotensin-converting enzyme inhibitors, angiotensin receptor blockers, statins, or calcium channel blockers was recorded. All procedures followed were in accordance with the Declaration of Helsinki and the ethical standards of the responsible committee on human experimentation (Taipei Veterans General Hospital). All patients signed consent forms before participation.

### Sample Collection and Biomarker Measurement

Peripheral blood was collected from each participant in ethylenediaminetetraacetic acid tubes. All samples were processed within 3 h of collection. The blood samples were centrifuged (3,000 × *g*, 10 min, 4°C) to remove cells and debris. The supernatants were transferred to ribonuclease-free tubes and stored at −80°C until analysis. Gal-3 and IL-6 levels were measured using Human ProcartaPlex Multiplex and Simplex kits (Thermo Fisher Scientific, Waltham, MA, USA).

### Statistical Analysis

Dichotomous variables were expressed as proportions; cases and controls were compared using the chi-squared test, whereas continuous variables were examined using the Mann–Whitney *U*-test to identify potential confounders. Associations with a probability of <0.10 were considered potential confounders and used in the multivariate analyses. The association between Gal-3 and IL-6 was studied using Spearman's correlation analysis. A multivariate logistic regression analysis was performed to assess the association between Gal-3 and the risk of AAA after adjustment for the identified potential confounders. These data analyses were performed using PRISM software (version 5.0, GraphPad, San Diego, CA, USA). A one-way analysis of variance with Tukey's *post-hoc* test was used to evaluate between-group differences. Statistical significance was defined as *p* < 0.05.

## Results

We performed a cross-sectional study to analyze plasma Gal-3 and IL-6 levels in both control patients (*n* = 195) and patients with AAA (*n* = 151). The demographic characteristics are presented in Table 1. Age, sex, hypertension, smoking, hypercholesterolemia, diabetes mellitus, peripheral vascular disease, and chronic obstructive pulmonary disease as well as the use of angiotensin-converting enzyme inhibitors, angiotensin receptor blockers, statins, β-blockers, and calcium channel blockers differed significantly between the two groups.

**Table 1:** Clinicodemographic characteristics of control patients and patients with AAA.

**Variables**	**Control (*n* = 195)**	**AAA (*n* = 151)**	***p*-value**
Aortic diameter (mm)	22.7 ± 3.7	60.6 ± 17.8	<0.0001
Age (years)	69.3 ± 9.9	78.5 ± 8.5	<0.0001
Sex (male/female), *n* (%)	107/88 (54.9/45.1)	137/14 (90.7/9.3)	<0.0001
Body weight, kg	64.9 ± 13.8	65.5 ± 12.8	0.720
Height, cm	163.0 ± 8.4	163.6 ± 7.3	0.625
Hypertension, *n* (%)	183 (93.8)	128 (84.8)	0.007
Smoking, *n* (%)	28 (14.4)	86 (60.0)	<0.0001
Hypercholesterolemia, *n* (%)	183 (93.8)	54 (35.8)	<0.0001
Diabetes mellitus, *n* (%)	57 (29.3)	17 (11.3)	<0.0001
Peripheral vascular disease, *n* (%)	7 (3.6)	14 (9.3)	0.039
COPD, *n* (%)	3 (1.5)	44 (29.1)	<0.0001
**Medications:**			
ACEi/ARB, *n* (%)	132 (67.7)	55 (37.7)	<0.0001
Statins, *n* (%)	83 (42.6)	22 (14.6)	<0.0001
β-blocker, *n* (%)	17 (8.7)	60 (39.7)	<0.0001
Calcium channel blocker, *n* (%)	44 (22.6)	60 (39.7)	0.001

*Values are presented as mean ± standard deviation unless otherwise specified. Abbreviations: n, number of patients; AAA, abdominal aortic aneurysm; COPD, chronic obstructive pulmonary disease; ACE, angiotensin-converting enzyme; ARB, angiotensin receptor blocker. A Mann–Whitney U test was used to compare continuous variables, and Fisher's exact test (two-sided) was used to compare categorical data*.

**Figure 1:**
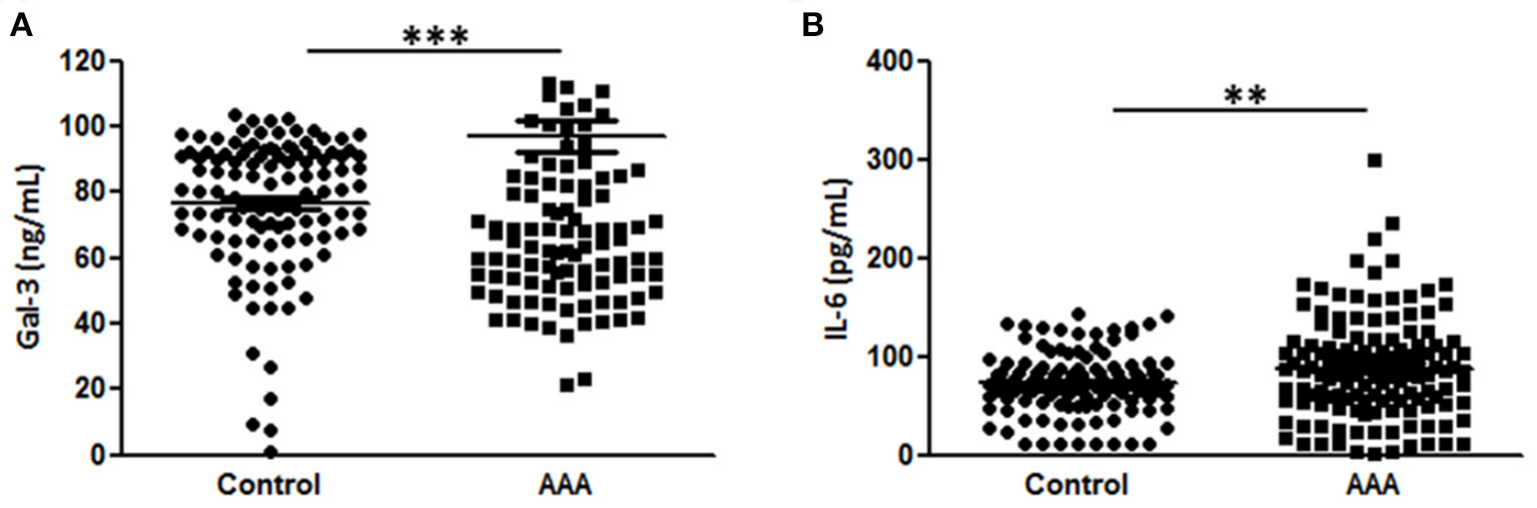
Diagnostic value of Gal-3 and IL-6. **(A)** Gal-3 and **(B)** IL-6 concentrations in plasma samples from patients with AAA (n = 151) and control patients (n = 195). Data are expressed as mean ± standard error of the mean. **p < 0.001, ***p < 0.001.

Plasma Gal-3 levels were significantly higher in patients with AAA than in control patients (96.9 ± 4.5L vs. 76.5 ± 1.9 ng/mL, Figure 1A). In this sample set, we analyzed the plasma concentrations of IL-6, which is reportedly associated with AAA (7, 19, 20). The levels of IL-6 were higher in AAA samples than in healthy control samples (92.8 ± 5.2 pg/mL vs. 72.5 ± 3.0 pg/mL; Figure 1B).

**Figure 2:**
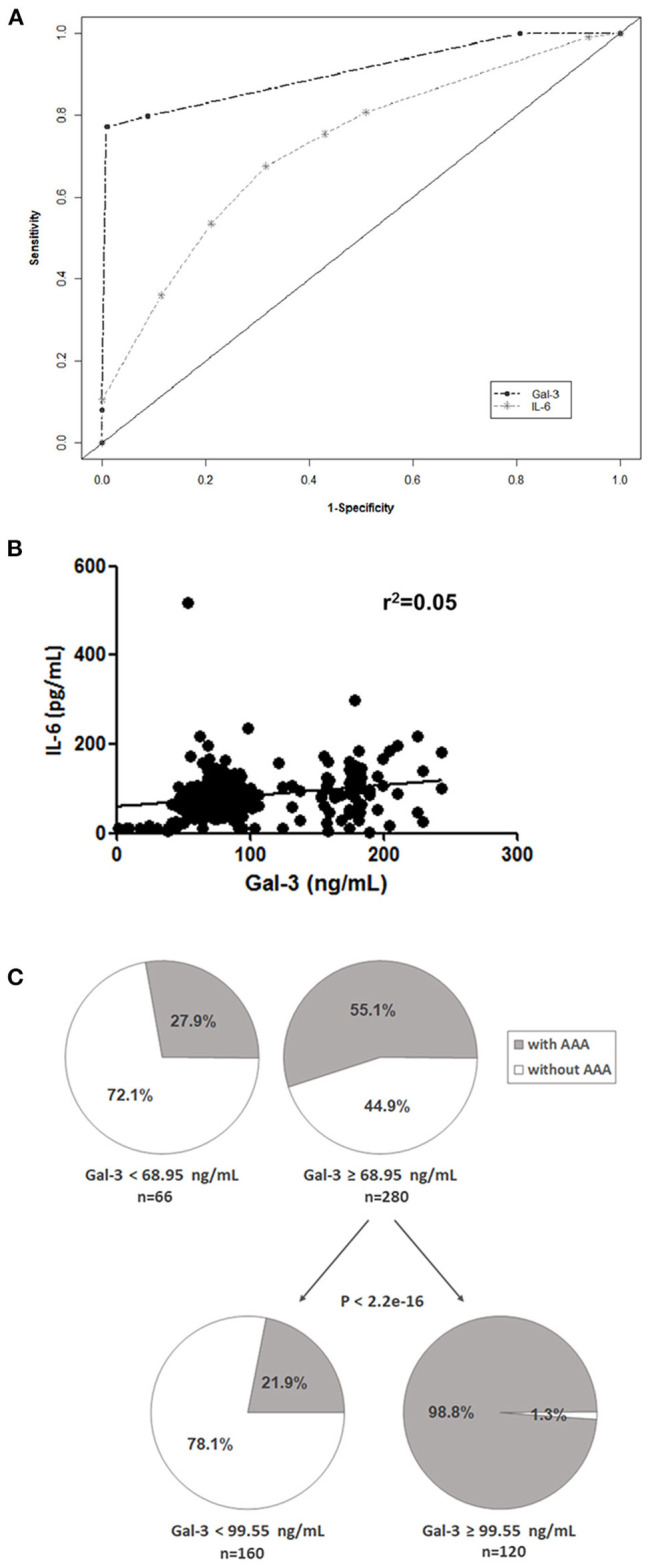
Potential prognostic value of Gal-3. **(A)** Receiver operating characteristic curves of Gal-3 and IL-6 were used to differentiate patients with AAA from control patients. **(B)** Correlation between Gal-3 and IL-6 levels in patients with AAA and control patients. **(C)** CART analysis with plasma Gal-3 levels as the independent variable and AAA as the dependent variable (all patients included).

We performed a classification and regression tree (CART) analysis and the diagnostic values of Gal-3 and IL-6 were evaluated using receiver operating characteristic curve analysis (Figure 2A). The results revealed that Gal-3 levels predicted AAA presence (area under the curve [95% confidence interval], 0.91 [0.83–0.92]) significantly more accurately than did IL-6 levels (0.72 [0.62–0.74]). A poor correlation was observed between plasma Gal-3 and IL-6 levels in patients with AAA (Pearson's r^2^ = 0.05, Figure 2B). Using AAA as the dependent variable by using CART analysis, we divided patients into two categories based on plasma Gal-3 levels: low (<68.95 ng/mL) and high (≥68.95 ng/mL; Figure 2C). The low Gal-3 group included 27.9% of patients with AAA, whereas the high Gal-3 group included 55.1% of patients with AAA. Furthermore, CART analysis divided the high Gal-3 group into two categories-those with Gal-3 concentrations of <99.55 ng/mL and ≥99.55 ng/mL. It is noteworthy that the 98.8% of patients with Gal-3 ≥99.55 ng/mL had AAA (*p* <2.2e-16).

To further examine the efficacy of Gal-3 for AAA diagnosis, we performed a logistic regression analysis after adjustment for age, sex, hypertension, smoking, hypercholesterolemia, diabetes mellitus, chronic obstructive pulmonary disease, and medication usage. The significant association between circulating Gal-3 levels and AAA persisted after adjustment for those factors (Table 2).

**Table 2:** Logistic regression analysis of Gal-3 for the presence of AAA.

**Variables**	**Univariate analysis OR (95%CI)**	***p*-value**	**Multivariate analysis OR (95%CI)**	***p*-value**
Gal-3	1.02 (1.01–1.03)	0.000	1.02 (1.00–1.04)	0.021
IL-6	1.00 (1.00–1.01)	0.002	1.00 (0.99–1.01)	0.851
Age	1.09 (1.06–1.12)	0.000	1.11 (1.05–1.17)	0.000
Male	12.38 (6.29–24.39)	0.000	11.79 (3.05–45.63)	0.000
Hypertension	0.41 (0.18–0.96)	0.39		
Smoke	7.4 (4.06–13.45)	0.000	10.27 (3.40–31.02)	0.000
Hypercholesterolemia	0.19 (0.11–0.34)	0.000	0.18 (0.06–0.55)	0.003
Diabetes mellitus	0.33 (0.17–0.63)	0.001	0.32 (0.10–1.07)	0.063
COPD	15.15 (4.56–50.33)	0.000	10.52 (1.80–61.39)	0.009
ACEi/ARB	0.26 (0.15–0.43)	0.000	0.16 (0.06–0.45)	0.001
Statins	0.24 (0.13–0.44)	0.000	0.25 (0.07–0.88)	0.030
β-blocker,	9.85 (4.28–22.66)	0.000	13.20 (3.40–51.23)	0.000
Calcium channel blocker	3.34 (1.85–6.04)	0.000	0.44 (0.15–1.27)	0.134

*A p-value < 0.05 in univariate analysis was included in the multivariate analysis. Gal-3, galectin-3; IL-6, interleukin-6; AAA, abdominal aortic aneurysm; COPD, chronic obstructive pulmonary disease; ACE, angiotensin-converting enzyme; ARB, angiotensin receptor blocker*.

**Figure 3:**
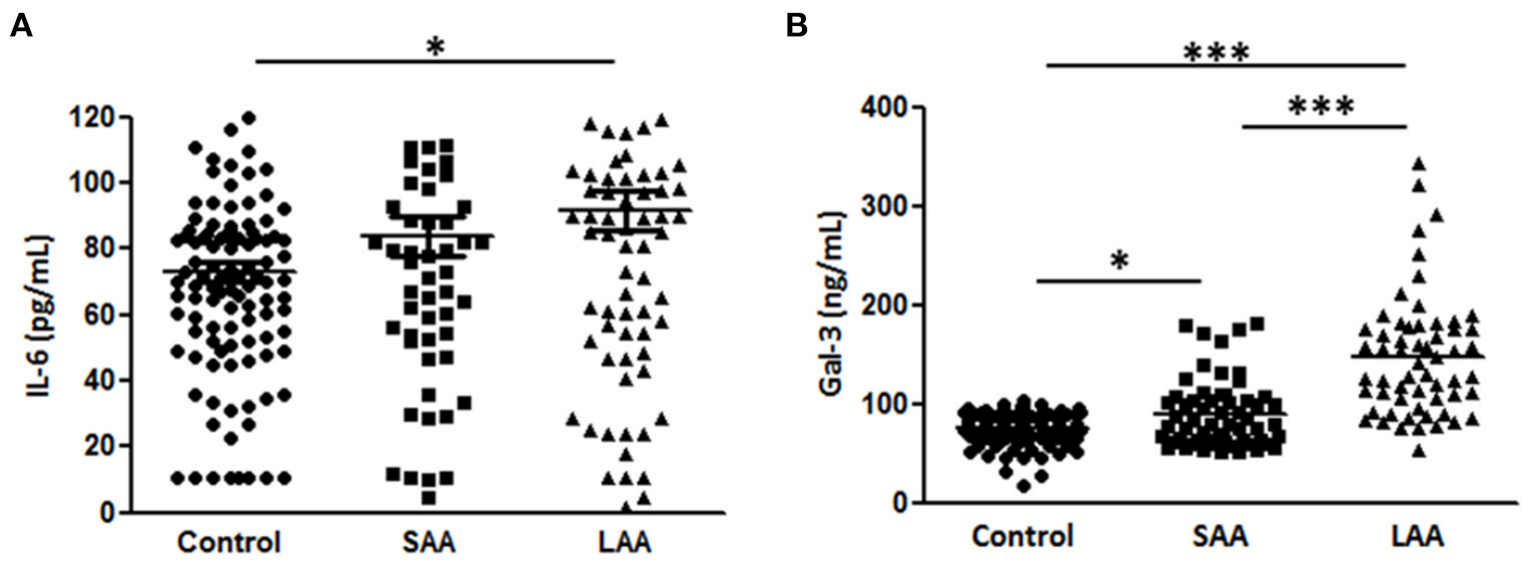
Gal-3 and IL-6 levels according to aneurysm size. **(A)** Gal-3 and **(B)** IL-6 concentrations in plasma samples from patients with a normal aortic diameter (control, *n* = 195), small aortic aneurysm (SAA, *n* = 58), and large aortic aneurysm (LAA, *n* = 93). Data are expressed as mean ± standard error of the mean. *p < 0.05, ***p < 0.0001.

Moreover, we analyzed the association of Gal-3 with aortic diameter as a surrogate marker of AAA evolution. Plasma Gal-3 levels were significantly correlated with aortic diameter after adjustment for potential confounding factors (Table 3). A subgroup analysis indicated that IL-6 levels were markedly higher in the LAA group compared with in the control group, but no significant difference was observed between the SAA and LAA groups (Figure 3A). However, Gal-3 was an independent variable for identifying subaneurysmal aortic dilatation and large aneurysms (Figure 3B).

**Table 3:** Multivariate linear analysis of Gal-3 for aortic diameter, including partial correlation coefficients.

**Variables**	**Standardized coefficients**	***t***	**Sig**.	**Correlations**		
	**Beta**			**Zero-order**	**Partial**	**Part**
Gal-3	0.132	2.156	0.032	0.290	0.136	0.101
Age (years)	0.178	3.155	0.002	0.414	0.198	0.147
Male, *n* (%)	0.267	5.169	0.000	0.395	0.314	0.241
Hypertension, *n* (%)	0.123	2.242	0.026	0.309	0.142	0.105
Smoke, *n* (%)	0.013	0.272	0.786	−0.072	0.017	0.013
Hypercholesterolemia, *n* (%)	−0.084	−1.471	0.143	−0.312	−0.094	−0.069
Diabetes mellitus, *n* (%)	−0.004	−0.089	0.929	−0.129	−0.006	−0.004
COPD, *n* (%)	0.038	0.724	0.470	0.280	0.046	0.034
ACEi/ARB, *n* (%)	−0.154	−3.029	0.003	−0.297	−0.190	−0.141
Statins, *n* (%)	−0.117	−2.047	0.042	−0.268	−0.130	−0.096
β-blocker, *n* (%)	0.210	4.177	0.000	0.370	0.258	0.195
Calcium channel blocker, *n* (%)	0.094	1.818	0.070	0.245	0.115	0.085
IL-6	−0.023	−0.381	0.703	0.161	−0.024	−0.018

*Gal-3, galectin-3; IL-6, interleukin-6; AAA, abdominal aortic aneurysm; COPD, chronic obstructive pulmonary disease; ACE, angiotensin-converting enzyme; ARB, angiotensin receptor blocker*.

## Discussion

The inflammatory process appears critical in the formation of AAAs, as demonstrated by extensive medial and adventitial inflammatory cell infiltration (21). Increased expression of proinflammatory cytokines is observed in aneurysmal tissue, and patients with AAA have elevated levels of circulating inflammatory cytokines (22, 23). Cytokines regulate the expression of MMPs, serine proteases, and cathepsin proteases, and local cytokine environments can drive aneurysm formation (24). Thus, circulation markers representing AAA pathology can be valuable for numerous reasons, such as in AAA diagnosis and prognosis. Although ultrasound is the gold standard for the diagnosis and surveillance of AAAs, with high sensitivity and specificity (25, 26), the frequency of ultrasound surveillance varies with aneurysm diameter. Furthermore, ultrasound is not recommended for patients with subaneurysmal aortic dilatation (27). Therefore, circulating biomarkers of inflammation, which reflect the aneurysmal size, can assist in the detection and prognosis of AAA (28). This study investigated the associations of two inflammatory markers (Gal-3 and IL-6) with aneurysmal size in patients with AAA and controls.

Patients with AAA have high levels of systemic inflammatory acute-phase reactants, such as hs-C-reactive protein, IL-1β, IL-6, interferon-γ, tumor necrosis factor-α, α-1-antitrypsin, orosomucoid, haptoglobin, and fibrinogen (5, 29–31). Furthermore, the cytokines IL-β, IL-6, IL-8, tumor necrosis factor-α, PGE2, and CCL2 were upregulated in AAAs compared with normal aortic samples (23, 31–35). Taken together, these findings have emphasized that inflammation is a crucial aspect of AAA pathogenesis. The aneurysmal growth rate is closely correlated with aortic size, with larger aneurysms having a higher growth rate (36, 37). A large population-based cohort study suggested that plasma Gal-3 concentration has a moderate positive association with AAA incidence (38). Our findings confirmed that Gal-3 is a specific marker of AAA. Gal-3 is likely a chemotactic molecule for macrophages (12). Thus, its expression could be associated with various cardiovascular diseases. The increased risk of AAA observed in patients with higher Gal-3 levels may reflect the recruitment of inflammatory cells, including activated macrophages, in the arterial system and the subsequent secretion of Gal-3. Moreover, Gal-3 expression is enhanced when macrophages or aortic vascular SMCs are loaded with lipids and transformed into foam cells. Therefore, Gal-3 can be a marker of vascular SMC phenotype switching (39).

A study revealed a positive correlation between aneurysm surface area and mean IL-6 level (Spearman's rank correlation *r* = 0.48; *p* = 0.003) (40). Furthermore, Flondell-Site et al. reported a significant correlation between AAA size and IL-6 levels (Spearman's *r* = 0.237, *p* < 0.0001) (41). However, Juvonen et al. observed no correlation between IL-6 levels and aneurysm diameter or expansion (22), and Lindqvist et al. identified no significant correlation between IL-6 levels and the maximum diameter of unruptured AAAs (42). In the present study, multivariate logistic regression analysis also failed to demonstrate a significant association between IL-6 levels and AAA. Therefore, the role of IL-6 in predicting AAA progression remains controversial.

This study has several limitations. Our sample size was not high enough to yield sufficient power for determining the clinical significance of these biomarkers. Information on the time between biomarker identification and disease outcome was not available in this cross-sectional study without prospective follow-up. Therefore, we could not reach conclusions regarding aneurysm progression. Furthermore, performing multiple comparisons is associated with a risk of false significance. However, we attempted to minimize this risk by using univariate and multivariate analyses.

## Conclusions

Circulating Gal-3 may be an independent variable for distinguishing between subaneurysmal aortic dilatation and large aneurysms. Further investigation is required to determine whether Gal-3 predicts aneurysm growth.

## Data Availability Statement

The original contributions presented in the study are included in the article/supplementary material, further inquiries can be directed to the corresponding author/s.

## Ethics Statement

The studies involving human participants were reviewed and approved by Taipei Veterans General Hospital. Reference number: 2016-07-013AC. The patients/participants provided their written informed consent to participate in this study.

## Author Contributions

H-YL performed the experiments. H-YL and C-MS analyzed the data. H-YL and S-HS interpreted the results of the experiments. H-YL and AW prepared the figures. H-YL and T-MC drafted the manuscript. Y-CL and C-CS edited and revised the manuscript. Y-CL conceived and designed the research. All authors approved the final version of the manuscript.

## Conflict of Interest

The authors declare that the research was conducted in the absence of any commercial or financial relationships that could be construed as a potential conflict of interest.
